# A Case of Confirmed Ceftriaxone-induced Immune Thrombocytopenia

**DOI:** 10.7759/cureus.4688

**Published:** 2019-05-17

**Authors:** Shady Piedra Abusharar, Neal Shah, Ravi Patel, Rohit Jain, Hyma V Polimera

**Affiliations:** 1 Internal Medicine, Penn State College of Medicine/ Penn State Health Milton S. Hershey Medical Center, Hershey, USA; 2 Internal Medicine, Penn State Health Milton S. Hershey Medical Center, Hershey, USA

**Keywords:** thrombocytopenia, immune thrombocytopenia, drug induced thrombocytopenia, meningitis, ceftriaxone

## Abstract

Drug-induced immune thrombocytopenia (DITP) is a rare, but potentially fatal cause of isolated thrombocytopenia. DITP is thought to occur when drug-dependent antibodies bind to the platelet membrane glycoproteins to activate platelet consumption signaling. Common implicated drugs include quinine/quinidine, penicillamines, valproic acid and cotrimoxazole. Ceftriaxone is a rare culprit with only six reported cases since 1991, of which only three were confirmed with drug-dependent antiplatelet antibodies. We describe a case of antibody confirmed ceftriaxone-induced immune thrombocytopenia after initiation of empiric antibiotic therapy for acute bacterial meningitis.

## Introduction

Acquired isolated thrombocytopenia is a common hematological finding in the inpatient setting [1]. Drug-induced immune thrombocytopenia (DITP) is a rare, but potentially fatal cause of isolated thrombocytopenia with a cited incidence of 10 in 1,000,000 cases [2]. It is understood to be most commonly caused by formation of drug-dependent platelet reactive antibodies which lead to the accelerated destruction of platelets [3]. It is considered to have a different pathogenesis from heparin-induced thrombocytopenia (HIT), where drug-associated antibodies promote thrombosis and platelet activation [4]. Unlike other causes of isolated thrombocytopenia, the platelet nadir in DITP is commonly observed to be less than 20,000/μL [3]. This typically occurs within one to two weeks of exposure to the inciting medication, and resolves within five to seven days of discontinuation [5]. Although it can occur with any drug, most commonly implicated drugs are quinine/quinidine, penicillamine, valproic acid, and cotrimoxazole [5,6]. The diagnosis is by exclusion and correlation of clinical and laboratory findings with drug initiation. Although not necessary, it can be confirmed by the presence of drug-dependent antiplatelet antibodies. Prompt discontinuation of inciting medication is key to resolution and prevention of serious complications, including GI and intracranial bleeding [7].

## Case presentation

A 71-year-old female with a past medical history of bilateral ductal carcinoma in situ, hypertension, and recent bilateral knee replacement presented to the emergency department with worsening headaches, confusion, photophobia, fevers and fatigue. Initial vital signs were temperature of 38.4°C, heart rate of 123 beats/min, blood pressure 164/97 mmHg and oxygen saturation at 98% on room air. Physical exam revealed lethargy, irritability and nuchal rigidity. Workup demonstrated WBC of 11,400/μL, Hgb of 10.8 g/dl, platelets 347,000/μL. Head CT was unremarkable. Lumbar puncture showed elevated nucleated cells with neutrophilic predominance. She was started on empiric therapy with vancomycin, ceftriaxone, trimethoprim-sulfamethoxazole (TMP-SMX, or bactrim) to cover for* Listeria* (penicillin was listed as an allergy) and acyclovir. Cerebrospinal fluid (CSF), herpes simplex virus (HSV), polymerase chain reaction (PCR) and culture returned negative, thus vancomycin, acyclovir and bactrim were discontinued after three days. Ceftriaxone was continued to complete a seven-day course. At this point, patient’s presenting symptomatology was resolving.

**Figure 1:**
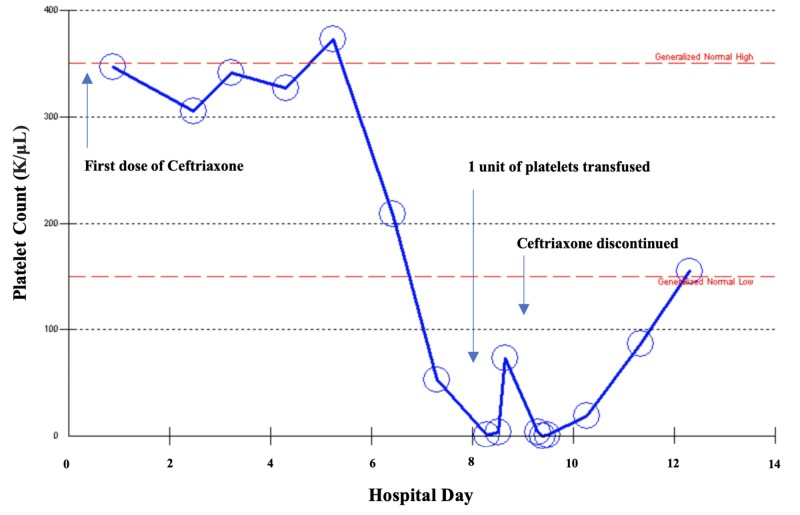
Trend of platelet count during hospital course highlighting causal relationship with initiation and discontinuation of ceftriaxone.

On hospital day six, platelets dropped to 1,000/μL. With 1 unit of platelet transfusion, platelets increased to 73,000/μL, but down trended to zero. Figure 1 illustrates the platelet count over the course of the patient's hospital stay. A peripheral blood smear showed zero platelets per high power field, with no noted schistocytes or platelet clumping. Immature platelet fraction was 30.7%, indicative of peripheral destruction with appropriate marrow response. Patient’s hemoglobin remained stable during her hospital course with no clinical evidence of bleeding. The patient received prophylactic heparin during hospitalization with a 4T score of 3, suggesting a low probability of heparin-induced thrombocytopenia. Vitamin B12 and copper were within normal limits. Given the timing and severity of thrombocytopenia after being on three antibiotics commonly associated (bactrim, vancomycin and ceftriaxone), DITP was suspected. Ceftriaxone was discontinued and she was started on dexamethasone 40 mg/day for four days. Platelets began to uptrend as can be seen in Figure 1. Platelet count at the time of discharge was 155,000/μL and 346,000/μL at a three-week follow-up. Post discharge, serum antibody testing returned positive for ceftriaxone-dependent platelet reactive IgG antibodies and negative for vancomycin and bactrim, further supporting the diagnosis of DITP secondary to ceftriaxone.

## Discussion

DITP is rare, and diagnosis requires exclusion of more commonly reported causes of thrombocytopenia. In this patient, the clinical picture was further complicated by presence of acute meningitis, and recent initiation of various new antibiotics. Thrombocytopenia can be inherited or acquired, and the etiology can be broadly classified into four categories which include pseudothrombocytopenia, platelet underproduction, peripheral destruction and splenic sequestration. Pseudothrombocytopenia should be ruled out early on, as should other serious peripheral destructive etiologies such as disseminated intravascular coagulation (DIC), thrombotic thrombocytopenic purpura (TTP), immune thrombocytopenia (ITP), and HIT. Common causes of platelet underproduction, such as nutritional deficiencies (vitamin B12, folate and copper), as well as bone marrow disorders should also be considered.

In this case, peripheral blood smear was unremarkable for schistocytes as seen in DIC or TTP, and labs did not indicate any vitamin deficiencies. In addition, a nadir below 1,000/μL did not support the diagnosis of HIT, as platelet count usually remains above 20,000/μL. ITP, like DITP, is also a diagnosis of exclusion, hence could not be ruled out. However, given convincing causal relationship of laboratory findings with initiation and discontinuation of ceftriaxone, DITP secondary to ceftriaxone seemed the most likely diagnosis. The diagnosis of DITP can be confirmed by detection of drug-dependent platelet reactive antibodies [7]. Recurrence with drug re-challenge also provides strong evidence of DITP, however due to risk of thrombocytopenia, this is rarely done. In our patient the diagnosis was confirmed by detection of ceftriaxone-dependent platelet reactive IgG antibodies.

Few cases in the literature report ceftriaxone-induced immune thrombocytopenia. A University of Oklahoma database, which tracks the number of DITP cases for single drugs, lists only six reported cases of ceftriaxone-induced immune thrombocytopenia in the literature from 1991 to 2013 [8]. Only three were confirmed with the presence of ceftriaxone-dependent antiplatelet reactive antibodies [7,9]. In two reported cases Grossjohann et al. demonstrated antibodies were found to react with platelets via epitopes residing on the GPIIb/IIIa subunit and GPIX complex [7]. This interaction likely results in the accelerated platelet destruction and ensuing thrombocytopenia. However, it remains unclear how ceftriaxone, or other implicated medications incite the formation of platelet reactive antibodies.

DITP occurs within one to two weeks after initiation of inciting medication, and resolves within five to seven days of discontinuation. Management of DITP requires prompt discontinuation of inciting drug. Platelet transfusion should be initiated if there are clinical signs of bleeding, or with platelet count <10,000/μL [9]. DITP can be difficult to distinguish from ITP, and hence immunosuppression therapy can be started. Intravenous immunoglobulin (IVIG) can also be considered if bleeding is severe.

Empirical treatment of acute meningitis requires prompt initiation of IV vancomycin and ceftriaxone. It often requires initiation of ampicillin in adults over 50 years of age to cover for *Listeria* *monocytogenes*. DITP secondary to penicillin, vancomycin and sulfonamides have been more commonly reported in the literature than ceftriaxone [2-5,8]. This is particularly important to consider in the setting of acute meningitis, where empiric treatment involves initiation of ceftriaxone, among other medications also known to cause DITP. In this case ceftriaxone was determined to be the inciting medication as all other antibiotics had been discontinued by hospital day three. Hence, this case highlights that ceftriaxone can in fact induce DITP, and emphasizes the importance of clinical correlation to determine the inciting medication.

Furthermore, this case points attention to a potential therapeutic challenge in patients who develop ceftriaxone-induced immune thrombocytopenia or who have a history of prior drug reaction in the setting of acute bacterial meningitis. In such cases alternative antibiotic therapy must be considered. Cefotaxime, a 3rd generation cephalosporin like ceftriaxone, in combination with vancomycin may be considered for treatment of acute bacterial meningitis. Our literature search showed no reported cases of cefotaxime-induced immune thrombocytopenia, hence this might be a suitable option. However, no literature was found addressing the risk of cross reactivity with 3rd generation cephalosporins in the context of DITP. Alternatively, fluoroquinolone such as moxifloxacin may be used in place of a 3rd generation cephalosporin [10]. However, data on the efficacy of fluoroquinolones is limited and should only be used in unusual cases or in patients who cannot tolerate standard anti-microbial therapy [11]. Hence achieving appropriate and effective antibiotic therapy in such cases may be challenging, and further highlights there is limited data to guide treatment in such circumstances.

## Conclusions

We present a rare case of confirmed ceftriaxone-induced immune thrombocytopenia after initiation of empiric treatment for acute bacterial meningitis. This case highlights the importance of maintaining a high clinical suspicion of DITP in the setting of unexplained thrombocytopenia. The culprit medication can be hard to identify in patients who are acutely ill and started on various new medications on admission. DITP in the setting of acute meningitis poses a therapeutic challenge, and may require alternative antibiotic therapy.
